# MSeqDR PMD-VR: An Expert-Curated Virtual Registry of 11,000 Mitochondrial Disease Cases Established Through Literature Mining and Generative AI Augmentation

**DOI:** 10.3390/genes17070757

**Published:** 2026-06-30

**Authors:** Lishuang Shen, Marie T. Lott, Elizabeth M. Mccormick, Colleen C. Muraresku, Kierstin Keller, Douglas C. Wallace, Zarazuela Zolkipli-Cunningham, Shamima Rahman, Marni J. Falk, Xiaowu Gai

**Affiliations:** 1 Pathology Informatics, Bioinformatics, and Data Science, Department of Pathology and Laboratory Medicine, Children’s Hospital Los Angeles, Los Angeles, CA 90027, USA; 2 Center for Mitochondrial and Epigenomic Medicine, Division of Human Genetics, Department of Pediatrics, Children’s Hospital of Philadelphia, Philadelphia, PA 19104, USA; lottm1@chop.edu (M.T.L.); kellerk@chop.edu (K.K.); wallaced1@chop.edu (D.C.W.); zolkipliz@chop.edu (Z.Z.-C.); falkm@chop.edu (M.J.F.); 3 Mitochondrial Medicine Frontier Program, Children’s Hospital of Philadelphia, Philadelphia, PA 19104, USA; mccormicke@chop.edu (E.M.M.); clarkec@chop.edu (C.C.M.); 4 Department of Pediatrics, Perelman School of Medicine, University of Pennsylvania, Philadelphia, PA 19104, USA; 5 Department of Genetics & Genomic Medicine, UCL Great Ormond Street Institute of Child Health, London WC1N 1EH, UK; shamima.rahman@ucl.ac.uk; 6 Department of Pediatrics, Medical College of Wisconsin, Milwaukee, WI 53226, USA; 7 Mellowes Center for Genomic Sciences and Precision Medicine, Medical College of Wisconsin, Milwaukee, WI 53226, USA

**Keywords:** mitochondrial disease, literature mining, human phenotype ontology (HPO), Generative AI (GenAI), large language model, Leigh syndrome spectrum (LSS), rare disease

## Abstract

**Background/Objectives:** Patient registries are essential for rare disease research, yet the extensive genetic and phenotypic heterogeneity of primary mitochondrial diseases (PMDs) makes traditional registry development slow and resource-intensive. We established the MSeqDR PMD virtual registry (PMD-VR) to address this gap through systematic literature mining and semi-automated data harmonization. **Methods:** The PMD-VR captures, standardizes, and harmonizes published case-level PMD data using a semi-automated curation pipeline. A data transformation framework maps heterogeneous raw data terms to standardized common data elements (CDEs). A generative AI (GenAI) platform leveraging large language models (LLMs), augmented by Human Phenotype Ontology (HPO) and external biomedical knowledge sources, accelerates data transformation and generates simulated clinical reports. **Results:** Currently, PMD-VR contains approximately 11,000 de-identified literature-derived cases, including over 2300 Leigh syndrome spectrum (LSS), 278 MELAS, and 300 CPEO cases. The pipeline mapped 872 heterogeneous terms to 102 standardized CDEs. Pathogenicity assessments were captured for variants in over 7900 cases, including 3800 with mtDNA pathogenic or likely pathogenic variants. Modes of inheritance were inferred for 5212 cases. PMD-VR has supported ClinGen Mitochondrial Diseases Gene Curation Expert Panel (Mito-GCEP) efforts, providing phenotyped evidence for 440 curated LSS cases across 113 PMD genes. **Conclusions:** PMD-VR is among the largest single PMD registries, offering a scalable, web-accessible platform for generating analysis-ready cohorts from the published literature. It represents a rich resource enabling comprehensive PMD characterization with unprecedented breadth of genetic and phenotypic knowledge.

## 1. Introduction

Primary mitochondrial diseases (PMDs) are a group of rare inherited diseases with a collective prevalence of at least 1 in 4300 individuals [1]. Leigh syndrome spectrum (LSS), a severe and typically pediatric-onset PMD phenotype that incorporates Leigh syndrome and Leigh-like disease, is the most common subtype that nonetheless remains exceedingly rare (1 in 34,000 individuals) in the general population [2]. The ad hoc creation of a comprehensive traditional registry for PMDs through active patient enrollment is challenging due to the low prevalence, difficulty in reaching diagnosis, high mortality rate, and need for intensive collaborative efforts across geographically dispersed populations. Consequently, most registries have historically been limited in size and geography to regional or national cohorts. The North American Mitochondrial Disease Consortium (NAMDC) Registry (https://namdc.rarediseasesnetwork.org), a significant joint effort in North America by 17 centers, is a physician-populated registry that enrolled 1553 participants between 2011 and 2018 [3,4]. Similarly, the Cure Mito Foundation started a global patient-populated registry for LSS cases, with a report on 113 enrolled cases in 2023 [5]. The Australian Genomics Mitochondrial Flagship reported 140 cases as of 2025 with 77 cases receiving a molecular diagnosis [6]. The UK Mitochondrial Disease Patient Registry (https://www.mitochondrialdisease.nhs.uk/for-clinicians/patient-registry/) is another notable registry, as is the United Mitochondrial Disease Foundation (UMDF) MitoShare patient-entered registry (https://umdf.org/mitoshare-registry/) that has recently surpassed 2000 individuals enrolled with self-reported mitochondrial disease across the spectrum of definite and suspected cases as well as their family members. A recent extensive literature review identified 17 articles describing 13 unique registries located in North America, Europe, Australia, and West Asia, with only half of the registries currently reported as active [7]. Such registries are often focused on clinical trial readiness and natural history data collection.

The Mitochondrial Sequencing Data Resource (MSeqDR) Consortium began in 2012 as a grassroots effort to organize the mitochondrial genetics clinical, diagnostic, and research community. Since its inception, MSeqDR consortium members have played a crucial role in advancing PMD research by developing and providing bioinformatics and genomic methods and resources, primarily through the open access web-based platform available at https://mseqdr.org [8,9]. MSeqDR’s mission is to enhance PMD knowledge through data sharing, standardization, and bioinformatics innovation. Recognizing both the difficulty in building ad hoc PMD registries and the wealth of information available in the published literature, the MSeqDR Consortium initiated in 2016 a project to build a semi-automated bioinformatics framework involving construction of a comprehensive virtual patient registry using its genomics and disease/phenotype data platforms. By leveraging the expertise of MSeqDR members in bioinformatics and data mining, MSeqDR aimed to create a large-scale virtual registry of publicly available PMD case-level data extracted in a semi-automated fashion directly from published studies. This effort was integrated with the NIH-funded ClinGen Mitochondrial Diseases Gene Curation (Mito-GCEP) [8,9,10] and Variant Curation Expert Panels (Mito-VCEP) [11].

Initial experience in building the virtual registry revealed that standardizing clinical data terms represents the most labor-intensive task. Ages and phenotypic descriptions in published cases are typically reported in unstructured narratives with inconsistent terminology and variable abbreviations in both term names and values, which require expert interpretation to commonly map to standardized data dictionaries like Human Phenotype Ontology (HPO) [12] and OMIM [13]. Traditional rule-based and manual curation approaches struggle with this inherent complexity, making curation efforts slow and error-prone for large-scale rare disease cohorts. Generative AI (GenAI) may overcome these limitations by leveraging the contextual understanding of large language models (LLMs) to interpret clinical descriptions, resolve ambiguous abbreviations, and accurately map unstructured text to standardized terms in a defined ontology. Here, we report a GenAI-empowered approach used to automate the most demanding tasks in data harmonization, followed by human-in-the-loop (HiTL) validation. Collectively, creation of the MSeqDR PMD virtual registry (PMD-VR), coupled with robust bioinformatics tools and the active adoption of GenAI and LLM technologies for knowledge extraction and transformation, supports accelerated PMD research efforts to improve understanding of these complex rare diseases.

## 2. Materials and Methods

**1.**  **Data Sources and Curation.**

**Figure 1:**
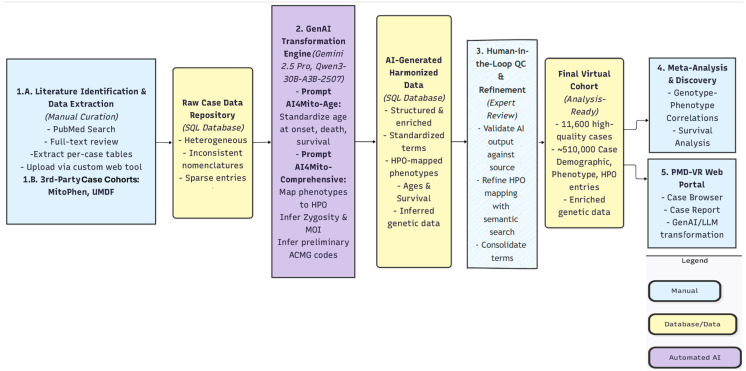
GenAI accelerated PMD-VR construction through data capture and transformation.

PMD-VR is web-based and database-driven. A hybrid approach is employed that combines semi-automated literature mining with manual expert curation to maximize the breadth and accuracy of data capture. Conventional bioinformatics methods and emerging LLM and GenAI methods are used in data collection, transformation, and reporting (Figure 1). The virtual registry has 3 primary data sources, as shown below.

**A. Semi-Automated Literature Mining:** A bioinformatics workflow was developed to facilitate extraction and importation of case-level data from the published literature. This workflow included several stages (Figure 1). First, the biocurator performed a PubMed search to identify publications reporting PMD cohorts with case-level data (Table 1). Second, data in tabular format and meta-information in free-text format were extracted from each publication’s main text and Supplemental Materials. Third, the tabular data were uploaded to PMD-VR via a web tool (https://mseqdr.org/clinical/msupload.php) for registry creation and case data upload (Supplementary Figure S1). During this process, raw column headers from each publication are mapped to standardized core data element (CDE) HPO terms through an interactive drag-and-drop interface (Supplementary Figure S1). Finally, all extracted data elements undergo manual review and refinement by expert curators from the ClinGen Mitochondrial Diseases Gene Curation Expert Panel (ClinGen Mito-GCEP, https://clinicalgenome.org/affiliation/40027/) to improve accuracy, completeness, and standardization. This workflow streamlined the process of capturing and curating a large volume of cases, with data stored to a backend MySQL database.

**B. ClinGen Mito-GCEP Dataset:** A dataset jointly curated with the NIH-funded ClinGen Mitochondrial Diseases Gene Curation and Variant Curation Expert Panels (Mito-GCEP/Mito-VCEP) was incorporated that comprised 440 biocurator phenotype-reviewed Leigh syndrome spectrum (LSS) PMD cases extracted from publications, including pathogenic variants in 113 nuclear and mitochondrial DNA (mtDNA) genes [10,11]. Additional processing remains underway of 1000+ published cases in the ongoing second phase of the Mito-GCEP project. The ClinGen Mito-GCEP focused on evaluating gene–disease associations for LSS in the first project period, and broader PMDs in the second project period, providing a high-quality, expert panel-reviewed consensus dataset for use in building the PMD-VR. The Mito-GCEP biocurators also contributed deeply characterized case data and expert-level gene–disease relationship (GDR) annotations, which were also ultimately incorporated into official ClinGen/ClinVar data releases [14].

**C. MitoPhen Database:** Through a data-sharing collaboration, a dataset of approximately 3600 PMD cases was integrated from the MitoPhen database (http://www.mitophen.org), which focuses on PMD phenotypes and mtDNA variant genotypes along with heteroplasmy-level data [15].

**2.**  **Case Data Capture and Processing** **.**

**Figure 2:**
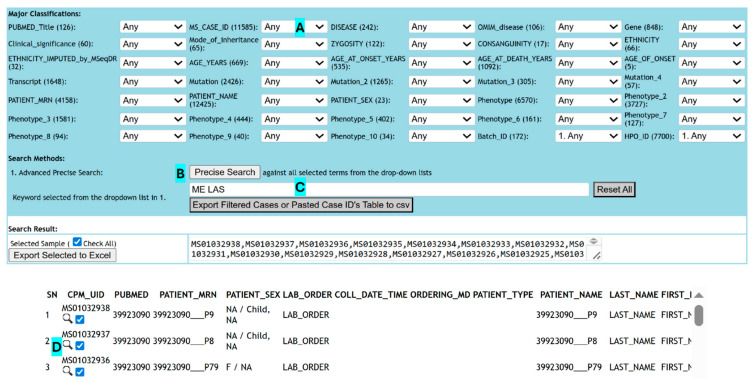
PMD-VR: Primary Mitochondrial Disease Virtual Registry Case Browser. (**A**) Directly select case by MS_XASE_ID from the PMD-VR, (**B**) “Precise Search” based on exact value match to any one or more of the 35 “Major Classification” data elements. (**C**) Google-style search by typing in the search boxes. (**D**) Right-click on the search icon to visit the per-case tabular report page (Figure 3).

The process of transforming the heterogeneous raw case data from diverse sources into a standardized and searchable virtual registry involves multiple steps. This is facilitated by existing in-house web-based tools and a structured database backend (Figure 2, Supplementary Figures S1 and S2). From 2017 to early 2025, data transformation primarily used conventional bioinformatics tools, as detailed below. More recently, emerging generative AI (GenAI) models from both open sources and paid commercial subscriptions have been leveraged for clinical and genomic data transformation and reporting.

### 2.1. Web-Based Clinical and Genomic Data Import

Data capture is conducted using an in-house web platform (https://mseqdr.org/clinical/msupload.php) with conventional bioinformatics tools (Figure 2, Supplementary Figure S1). The system is designed to accommodate heterogeneous tabular data and case-level demographic, clinical, and genomic data to be imported, regardless of the initial column organization, naming conventions, or number of columns. To streamline the common data element (CDE) harmonization, the platform’s drag-and-drop interface supports users in mapping imported raw column names to standardized core data fields, “MSeqDR Core Data Columns”, as in Supplementary Figure S1. This intuitive feature simplifies the alignment of diverse data structures. Beyond literature reports, the platform also supports tabular data import from other sources, such as lab or clinic datasets, where the project IDs are used in lieu of PubMed IDs, broadening the scope of data integration.

### 2.2. Data Harmonization to Standard Dictionaries

Data standardization ensures consistency and enables effective registry querying and in-depth analysis. Standardization is achieved through two methods. First, during data import, the drag-and-drop interface provides initial term mapping for standardization, as described above and in Supplementary Figure S1. Second, post-import, custom SQL scripts are run to systematically map the heterogeneous raw data terms and values to standardized dictionary terms and values stored in the backend MySQL database (Supplementary Figure S2). This involves converting and inferring phenotypes, disease names, and gene/variant terms/values, respectively, to controlled vocabularies such as HPO and OMIM, and standard gene/variant nomenclatures. These bioinformatics methods were the primary methods used to build PMD-VR to its status, and GenAI-enabled transformation is actively under exploration to enhance PMD-VR moving forward, as detailed below.

### 2.3. PMD-VR Case Browser

The PMD-VR case browser (https://mseqdr.org/virtualregistry.php) provided a high-level summary of the cases, grouped by over 30 core data elements (CDEs) as “Major Classification” (Figure 2). Each key data element is summarized as a drop-down list, which lists case numbers for each value group reported. Selecting a value from the drop-down list will pull the matching case entries into a list in the bottom table for quick access. A more flexible and responsive search can be conducted using one of two strategies:

**A.** “Google-style” Quick Fuzzy Search: Leveraging Ajax and jQuery, the PMD-VR platform offers a quick, “Google-style” fuzzy search across any of the over 30 CDEs, allowing users to quickly identify potential case matches based on partial or exact terms while typing in the search box. The tool lists all matching samples in a pop-up list. Each entry includes a brief description, with a single click leading to the detailed single case report for in-depth review and further crowdsourcing-style curation, and GenAI tool calling, as detailed in Section 2.4.

B. Precise Filtering: Users can refine the search using single-feature filters (gene name, phenotype, age, ethnicity) or a composite filter by combining multiple categories to create complex, precise queries based on specific combinations of clinical, genetic, and demographic criteria. Over 30 categorical clinical features are summarized into drop-down lists for users to select one or more features to compile their preferred filtering criteria.

### 2.4. Single Case Curation and Reporting

**Figure 3:**
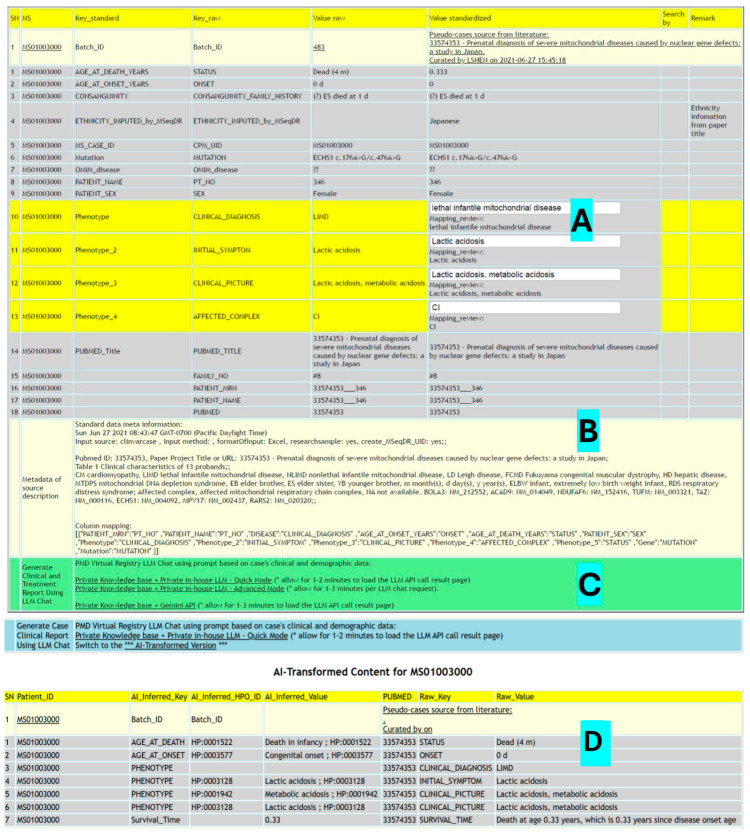
PMD single-case tabular report example for MS01003000. (**A**) Phenotype entries can be curated through this web page; (**B**) cohort and case metadata: PubMed, table name in the literature, and JSON of column mapping as “raw term name”/“standardized term name” pairs for defining the rule for core data element (CDE) mapping from the raw terms; (**C**) GenAI-based clinical report generation with in-house or external (Gemini) LLMs; (**D**) AI-transformed content for MS01003000.

Selecting a case from the pop-up list on the case browser or clicking a case link from query results opens a detailed single case report and curation interface (Figure 3). This interface presents all stored case data in a comprehensive tabular format, displaying both the standardized terms and values, along with their original source terms, for transparency and traceability. Some case data elements are displayed in editable text boxes, allowing for continuous curation and refinement. Edits are automatically saved under each user’s account and displayed alongside contributions from other users to support “crowdsourcing” curation by MSeqDR mitochondrial disease experts.

## 3. GenAI-Empowered Data Transformation and Analysis

### 3.1. GenAI Integration and In-House GenAI/LLM Platform Architecture

To overcome the limitations of labor-intensive manual curation and conversion, we initiated development of a novel, scalable framework for case data harmonization powered by GenAI. This framework is built upon a local platform prototype that integrates a private, PMD-focused knowledge base to augment the reasoning capabilities of LLMs. The platform utilizes a dockerized instance of AnythingLLM version 19.0 [16] as its primary web interface, which interacts with and manages various LLMs, either served locally via Ollama instance version 0.13.1 [17] or served by remotely calling subscription-based external LLM APIs.

### 3.2. Large Language Models (LLMs) and Knowledge Base

Our platform utilizes a hybrid of both locally hosted open-source models and state-of-the-art subscription-based APIs. The optimal models vary by specific task to balance performance, cost, and privacy considerations.

- **Open-Source Models:** For experimentation and baseline performance, and for maximal privacy consideration, we integrated several models to run locally, including the Qwen3 series (Qwen3-30B-A3B and Qwen3.6-35B-A3B) [18] and Google Gemma 3 (14 B) [19].
- **Subscription-Based APIs:** For production-level tasks requiring maximum accuracy and scalability, we integrated leading commercial APIs, including Google Gemini 2.5 Pro and Flash (Google AI Gemini API: https://aistudio.google.com/) [20] and OpenAI GPT-4o (OpenAI API: https://platform.openai.com/) [21].
- **Knowledge Base:** The platform’s reasoning is augmented by a local knowledge base built on the LanceDB vector database. Text embeddings for semantic indexing are generated using Google’s Gemini Text Embedding Model (text-embedding-004). This model was specifically chosen to ensure optimal compatibility with the Gemini and Gemma LLMs used in the primary data transformation workflow. The local knowledge base was populated with key biomedical ontologies (OMIM, HPO), essential for genetic disease research, and the curated, internally harmonized case data from the PMD-VR.

### 3.3. GenAI Platform Optimization and Prompt Engineering

Systematic evaluation of different embedding models and LLM combinations is ongoing to identify optimal pairings that maximize accuracy for retrieval-augmented generation (RAG) tasks. To ensure the reliability of the output in a critical medical context, key model parameters, such as “temperature,” are being optimized to a lower value (e.g., 0.1–0.2) to prioritize factual accuracy and minimize the risk of model fabrication (hallucination).

A structured prompting strategy was engineered to guide the data transformation process. The core prompt assigns GenAI the role of an “AI expert system emulating a board-certified clinical geneticist” and establishes strict guiding principles to ensure data fidelity. Specific prompts were designed for distinct tasks. For standardizing age-related data, a prompt, AI4Age, is designed to extract and convert age at reporting, age at onset, and age at death from various formats into a unified numerical value (in years), map these values to the corresponding HPO terms, and calculate survival time based on the available data.

Another prompt, AI4Report, is engineered to mimic clinical-grade report generation to enhance the readability and information for case reporting. It automates “clinical visit” virtual report generation by transforming the registry’s structured, tabular case data into human-readable, narrative clinical visit-style notes for the selected case. Reports are automatically standardized with HPO and OMIM term mappings based on case data and our local knowledge base. The report can also be used to include literature citations for the user to review that suggest GenAI-generated treatment and management recommendations, including summaries of potential treatment, management, and genetic counseling strategies by synthesizing information from the local case data, the knowledge base, and validated external web sources. A prominent disclaimer is appended to all such outputs, clarifying that they are for research purposes only and do not constitute clinical guidance.

## 4. Hardware and Infrastructure

### 4.1. PMD-VR Hosting

PMD-VR, accessible via the MSEQDr portal (https://mseqdr.org), is hosted on an Amazon Web Services (AWS) Elastic Compute Cloud (EC2) instance. This instance is configured with 8 vCPU threads and 16 GB of RAM [AWS EC2: https://aws.amazon.com/ec2/].

### 4.2. In-House GenAI/LLM Platform Hardware and Software

A dedicated local AI platform for GenAI/LLM experimentation and application development is hosted on local workstations. The primary system is a Lenovo Legion 7 laptop featuring an Intel Ultra 275k CPU (24 cores, 24 threads), 64 GB DDR5 RAM, and an Nvidia RTX 5090 GPU with 24 GB VRAM. The secondary system is a Dell XPS 17 laptop with an Intel Core i7 CPU (8 cores, 16 threads), 32 GB DDR4 RAM, and an Nvidia RTX 2060 GPU with 6 GB VRAM. Both workstations run the Ubuntu 24.04 LTS operating system [Ubuntu: https://ubuntu.com/]. The platform provides dedicated processing power for interfacing with local and subscribed LLMs, generating local text embeddings for input prompts, and hosting a local PMD knowledge base with web frontend.

## 5. Results

### 5.1. Case and Phenotypic Data Content

**Figure 4:**
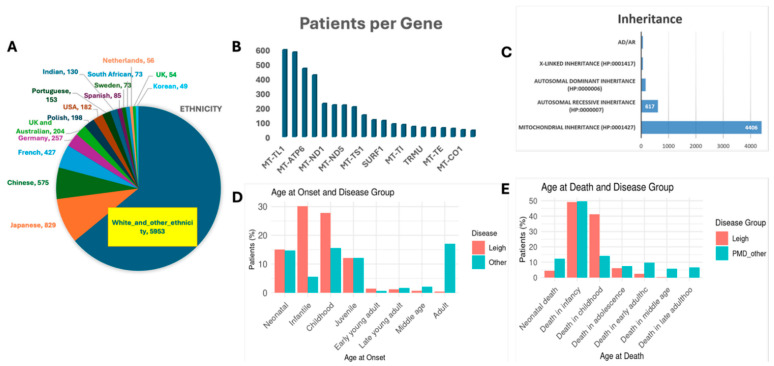
Demographic, clinical, and genetic characteristics of PMD cases in PMD-VR. (**A**) Ethnicity and country origin distribution of PMD cases, based on ethnicity information inferred via MSeqDR curation. (**B**) Age-of-onset distribution shows higher numbers of cases associated with mtDNA genes than nuclear genes in PMD cohort. (**C**) Numbers of cases assigned as mitochondrial inheritance, autosomal recessive, autosomal dominant, and X-linked. (**D**) Age at onset and (**E**) Age at death distribution showing higher early-life presentation in LSS compared with non-LSS PMDs.

PMD-VR development was initiated in 2016 with an initial cohort of 223 pediatric PMD patients [22]. It expanded significantly between 2018 and 2021, during which 2198 cases were captured, with a focus on LSS published cases. The registry served as a bioinformatics and data curation resource for the ClinGen Mito-GCEP expert panel, providing case-level genetic evidence for the classification of genes associated with LSS [10]. Rapid expansion continued from 2022 to 2025, with the iterative addition of 4470 (in 2022), 477 (in 2023), 2313 (in 2024), and 2287 (in 2025) cases. As of August 8, 2025, the MSeqDR PMD-VR had accumulated over 11,000 de-identified patient case entries from over 20 countries, making it one of the largest PMD case registries worldwide (Figure 2 and Figure 4).

The PMD cohort sizes in each source publication vary widely, from a few to hundreds of cases, with the MitoPhen compendium project being a notable exception. MitoPhen compiled thousands of cases from multiple original publications [12]. Several publications involving larger cohorts are listed in Table 1. The ~11,000 unique case entries in PMD-VR are curated from multiple data sources: ~8000 from in-house literature mining (data sources A and B), and 3696 cases from data source C, shared courtesy of the MitoPhen dataset [15]. The combined raw data and MSeqDR-inferred data entries total ~500,000, averaging ~44 data entries per case (Table 1). This dataset includes 1308 raw clinical and demographic terms, 872 of which have been mapped to 102 standardized terms to enhance data harmonization.

Due to the initial focus on LSS cases in Phase 1 of ClinGen Mito-GCEP, the PMD-VR captured 2306 LSS cases (OMIM: #256000), mostly from semi-automated literature mining and another 440 from Mito-GCEP expert curation review. Beyond LSS, ongoing disease name standardization has been completed for another ~2000 of the in-house curated PMD cases sourced from the published literature (excluding MitoPhen cases), mapping 130 distinct disease names. Major PMD phenotypic subsets include significant cohorts of: ~300 cases of Chronic Progressive External Ophthalmoplegia (CPEO) (OMIM: #157640, #590060), 278 cases of Mitochondrial Encephalomyopathy, Lactic Acidosis, Stroke-like Episodes (MELAS) (OMIM: #540000), and 1263 cases of Leber Hereditary Optic Neuropathy (LHON) (OMIM: #535000).

Among the 11,000 cases, 10,623 (97%) have at least one phenotype recorded, including 278 cases with ≥30 phenotype fields, reflecting the existence of deeply phenotyped cohorts. Age of onset is recorded for 7402 cases, and age at death for 2081 cases. For evaluation of causal genes and pathogenic variants, genetic variation data is available in PMD-VR for 7628 cases, linked to 2099 pathogenicity-assessed variants, including 5397 cases with a second reported variant. It should be noted that some “second variants” may represent alternate notations (protein or cDNA) of the same primary variant, so further curation is required to determine the precise number of cases with two or more distinct variants. Many of these variants have undergone pathogenicity assessment following ACMG guidelines according to the source literature.

Additional key metadata fields include consanguinity (633 cases), ethnicity (958 cases), mode of inheritance (4823 cases), zygosity (818 cases), and de novo vs. inherited status (519 cases).

### 5.2. Clinical, Demographic, and Genomic Data Transformation: The Conventional Bioinformatics Strategy

The greatest challenge in building a searchable virtual case registry from original publications was harmonization and standardization of the heterogeneous case data encoding terms used by the authors. This was conducted through a two-pronged approach. First, a web-based data import interface supported drag-and-drop column name standardization (Supplementary Figure S1), followed by helper tools for mapping free-text or abbreviated phenotypes to HPO terms (Supplementary Figure S2). Second, data were curated by experts using backend MySQL database scripts. These scripts, designed with clinical and genomic domain knowledge, mapped highly heterogeneous raw data to the disease, phenotype, gene, and variant databases underlying the MSeqDR data platform. This involved mapping the free-text phenotypes, disease names, and gene/variant terms to controlled vocabularies such as HPO, OMIM, MONDO, and standard gene/variant nomenclatures.

The data transformation standardized fields such as OMIM diseases, genes, variants, phenotypes (HPO terms), inheritance mode, zygosity, consanguinity, sex, age at onset/death, and ethnicity. From the original publications, 1321 raw table columns were identified, of which 872 were standardized into 102 common CDEs. As evident from the key mapping, this process can be complicated; for instance, the single standardized element “Disease” was mapped from over two dozen different raw field names used by the original authors. Additionally, when key data elements were not explicitly reported in the original tables, an MSeqDR curator from the ClinGen Mitochondrial Diseases Gene Curation Expert Panel would manually infer the information from the full text of the publication, which would then be added into the dataset with the designation “Inferred by MSeqDR”.

In addition to data element names, data values were also converted and standardized by a hybrid strategy combining manual and bioinformatics methods. For example, 186 raw disease names used by original authors were reviewed and mapped to 103 standardized OMIM disease terms. Similarly, age information originally presented in diverse day/week/month/year combinations was converted into a standardized format of year decimals.

### 5.3. Clinical Phenotypes to Human Phenotype Ontology (HPO) Mapping—The Conventional Bioinformatics Strategy and GenAI Strategy

Heterogeneous clinical and demographic data were mapped to over 100 “standard” clinical, biochemical, and genetic feature terms including HPO and OMIM disease terms. One central task was mapping the case raw phenotypes to standardized dictionaries. Mapping was conducted with conventional bioinformatics web tools (Supplementary Figure S2) that were based on candidate scoring and ranking according to the semantic similarity between the raw phenotype texts against HPO name/Synonym/Definition. If the case data phenotype used abbreviations, and if the keys were included in the cohorts’ metadata, such abbreviation keys were used for expanding the clinical terms into full-length values before feeding the semantic similarity-based mapping to the HPO dictionary (Supplementary Figures S2 and S3).

While HPO mapping is only partially completed, 4397 cases have already been completed with two or more HPO terms mapped. The most common phenotypes are skeletal muscle atrophy (HP:0003202), increased serum lactate (HP:0002151), global developmental delay (HP:0001263), intellectual disability (HP:0001249), and abnormal basal ganglia morphology (HP:0002134).

Through data transformation, inheritance modes for nearly 6000 cases have been inferred, including 3699 cases with maternal (mitochondrial DNA) inheritance (HP:0001427), 498 for autosomal recessive inheritance (HP:0000007), 314 with autosomal dominant inheritance HP:0000006), and 104 with X-linked inheritance (HP:0001417).

### 5.4. Preliminary Summary/Insight on PMD Cohort’s Demographic, Clinical, and Genetic Characteristics

Regarding age characteristics in the PMD-VR cohort (Figure 2 and Figure 4), disease onset occurred predominantly in early childhood, with 26.7% of cases presenting within the first year of life and 45.0% manifesting by five years of age. Compared with other PMDs, LSS exhibited a markedly earlier onset profile: LSS patients were 5.5-fold more likely to present as infantile onset (HP:0003593; 30.1% vs. 5.55%) and 5.8-fold less likely to present as adult-onset (HP:0003581; 3.67% vs. 21.33%). Mortality (AGE_AT_DEATH) patterns are similar to these onset trends, where 95.0% of reported LSS case deaths occurred before or during childhood as compared with 76.0% in non-LSS PMDs. However, caution is warranted in broadly applying this to LSS prognosis given the high potential for bias in reporting on death in published cases.

Genetic analysis demonstrated an over-representation of maternal (mitochondrial DNA) inheritance and associated genes in LSS relative to the broader PMD cohort (Figure 4). Among nuclear-encoded cases, autosomal recessive inheritance was the most frequent mode, exceeding the prevalence of both autosomal dominant and X-linked inheritance patterns. These findings highlight the strong association of LSS with early-onset, recessively inherited pathogenic variants and their significant impact on survival outcomes. However, they may well overestimate mortality based on potential bias toward reporting more severe cases and not reporting subsequent cases for known genes or variants in the published literature.

### 5.5. Comparison of Registry Size with Published Registries and Cohorts

Compared to traditional patient registries, the MSeqDR PMD-VR, encompassing over 11,000 literature-derived cases, represents a substantial new resource for users to exploit to facilitate future mitochondrial disease research. While direct granular comparisons are limited by variations in registry scope, data availability, and reporting practices, the MSeqDR PMD-VR registry’s large scale is informed by comparing the number of cases for specific diseases available in the PMD-VR versus in other registries.

For the more “common” PMDs and cases, the MSeqDR registry contains approximately 2306 LSS, 300 CPEO, and 278 MELAS cases. The Cure Mito Foundation global patient registry for LS reported 113 cases in 2023 [5]. The NAMDC registry, the result of an NIH-funded joint effort by 17 centers between 2011 and 2018, held 97 LS, 37 CPEO, and 71 MELAS cases among its 1553 cases [4]. Regarding cases with molecular diagnosis, 7738 of 11,000 cases in the PMD-VR carry pathogenicity-assessed genetic mutations. In comparison, the NAMDC cohort had 414 participants carrying pathogenic mtDNA variants, and 252 participants carrying pathogenic nuclear gene variants [4]. The Australian Genomics Mitochondrial Flagship reported 140 cases as of 2025 including 77 cases who had received molecular diagnosis [6]. These examples highlight the cost and burden of scaling PMD registries using traditional methods, and the advantage of literature mining to discern reported cases, like in PMD-VR.

## 6. GenAI-Empowered Data Transformation and Clinical Insights

### 6.1. LLM Selection and Performance in Case Data Transformation

After preliminary evaluation of several state-of-the-art LLMs for local deployment, a Qwen3-30B model quantized by TeichAI was selected from Huggin Face (URL: https://huggingface.co/TeichAI/Qwen3-30B-A3B-Thinking-2507-Claude-4.5-Sonnet-High-Reasoning-Distill-GGUF, Q4_K_M quantization). It was chosen as the primary engine due to its open-source nature and its balance of performance and moderate resource requirements. This model fits entirely within the VRAM of consumer-grade RTX-5090 (24 GB), achieving speeds of up to 110 tokens per second for clinical visit report generation and age data transformation tasks. It also runs on RTX-2060 GPU with 6 GB VARAM and 32 GB system RAM, albeit at significantly lower speed. Case reports generated using this model demonstrate similar structure and comparable content quality to those produced by Gemini 2.5 Pro in qualitative spot checking.

Google’s Gemini 2.5 Pro was selected as the engine for more complex data transformation tasks due to its superior performance in accuracy, speed, and ability to handle large context windows of up to 1 million tokens. The capability of this approach was demonstrated using a complex test case whose phenotype was described in unstructured, multi-symptom strings. Gemini 2.5 Pro successfully disaggregated raw text and correctly mapped more identifiable phenotypes to their corresponding HPO terms and IDs (Supplementary Table S5). In contrast, other models, including ChatGPT-4o and several open-source options, exhibited limitations, such as failing to disaggregate all terms from the unstructured text note or generating a higher rate of invalid HPO IDs.

Given the clear initial performance gap between models, we focused on optimizing the workflow around locally hosted open-source Qwen3 models for privacy and cost-sensitive tasks and using closed-source Gemini models for occasional complex tasks; both were always coupled with a human-in-the-loop review process.

### 6.2. A GenAI-Empowered Framework to Overcome Data Heterogeneity

A primary challenge in constructing the PMD-VR was the extreme data heterogeneity identified across published cohorts. The raw dataset contained over 1320 unique column headers; the conventional bioinformatics methods were used to map 872 of these data elements into 102 CDEs, through a conventional bioinformatics process that was labor-intensive and technically difficult. Furthermore, the encoding of clinical and genomic values was also highly inconsistent and reported in different formats and nomenclatures, necessitating data conversion and standardization before comprehensive meta-analysis could be attempted. Therefore, a GenAI-empowered framework was developed to address these challenges by simulating the reasoning of a human curator to standardize these complex data into human-friendly expressions without rigidly enforcing each regular expression rule. Results show that this strategy reduces the time and effort burden compared to the conventional semantic and string similarity-based methods. This is most evident in expanding abbreviations to their full terms (Figure 3, Supplementary Figure S3).

### 6.3. AI4Age Application in Age Data Transformation and HPO Mapping

As age data standardization is critical to characterize disease course, it was used as the first GenAI application in PMD-VR curation. AGE_AT_ONSET_YEARS and AGE_AT_DEATH_YEARS have 30 and 19 raw column headers, respectively (Supplementary Table S1). In addition, the values are encoded in multiple formats of pure numbers or year/month/day combinations. Together, they posed a major challenge in our previous curation effort for semi-manually transforming age data based on rigid bioinformatics rules. For age-related data transformation, a fine-tuned GenAI prompt achieved high concordance with manual curation judgment by ClinGen Mito-GCEP curators on the testing dataset when running with local deployment of the Qwen3-30B model, and achieved a speed of 10 cases per minute. Survival time is inferred after the age data standardization. The report is standardized into tables with six input columns and six inferred columns including years in number, HPO terms and data transformation rationales, as detailed in Supplementary Table S4.

### 6.4. Application in Phenotype-to-HPO Mapping

**Figure 5:**
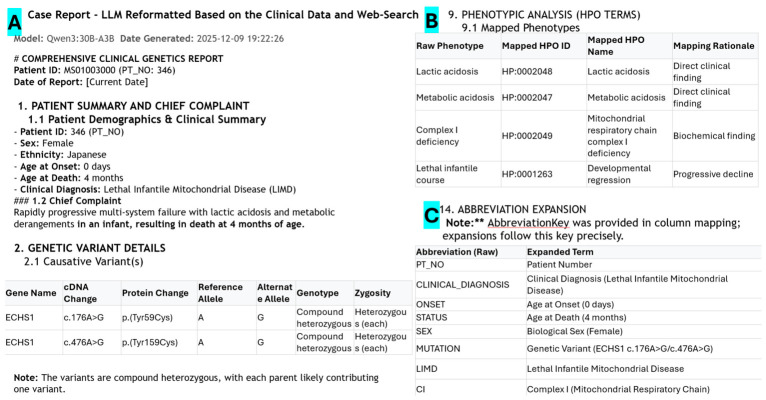
Generative AI–empowered case clinical report via local private Qwen3-30B LLM model and knowledge base. (**A**) Patient (MS01003000) demographics, clinical summary, and genetic findings; (**B**) HPO mapping from raw phenotypes; (**C**) abbreviations are mapped and expanded to full terms based on provided keys in case metadata and LLM foundation model.

A major task for building PMD-VR involves mapping raw clinical phenotype descriptions to standard HPO terms. The GenAI models greatly accelerated this process, accurately mapping raw phenotypes to HPO term names as was particularly evident in expanding abbreviations into full terms, and its reasoning capability in free-text mapping to HPO terms (Figure 3 and Figure 5B,C, Supplementary Figure S3, Supplementary Material S4). Yet, the models also demonstrated a tendency of “hallucination” especially when assigning specific HPO IDs, as observed in example case MS01003000, where GenAI did not always assign correct HPO IDs to the correctly transformed HPO terms (Figure 5). Generally, HPO term mapping performance varied with the LLM model, the case context, and prompt instruction, which called for more systematic optimization. To overcome the issue, the HPO mapping process incorporated the human-in-the-loop manual review validation (Figure 1) which combined the semantic similarity-based HPO term mapping tool used in a previous clinical production pipeline [23], and the LLM prompts were fine-tuned from a GenAI pipeline that previously achieved over 90% accuracy using Gemini-pro-2.5 [24].

We also experimented with GenAI-empowered term conversion and standardization on more terms regarding genes, clinical manifestations, medical terminology, and genomic feature nomenclature cross-mapping. Initial results are promising but require further improvement as well as quality review by human experts. Optimization of prompt engineering is underway to expand GenAI-based data transformation to backend data processing and standardization (Figure 3).

These examples highlight the need for a hybrid approach, combining the rapid text-processing strengths of GenAI with rigorous human-in-the-loop validation using conventional bioinformatics tools.

### 6.5. Ai4report Preliminary Automated Generation of “Clinical Reports”

In the per-case tabular report pages, LLM API call links are within the “Generate Clinical and Treatment Report Using LLM Chat” section (Figure 3C). Users may run one of the following tasks for AI-generated reporting: “Private Knowledge base + Private in-house LLM-Quick Mode” and “Private Knowledge base + Gemini API” (Figure 3C). Using a privacy-preserving in-house LLM platform, the local instance of the Qwen3-30B and Qwen3.6-35B models can generate a case report in under one minute. The “Clinical Reports” detailing PMD-VR cases that met user-specific search criteria (Figure 5 and Supplementary Material S4) are generated using a sophisticated LLM prompt named AI4Report, which was designed to transform the sparse, tabular per-case data into comprehensive, human-readable clinical visit-style reports. This prompt instructs the LLM to first transform the case data, then structure the output consistently across cases. AI4Report draws on the AI-transformed data and further augments it with information from an internal knowledge base and external literature searches of PubMed or general web searches. The resulting reports are divided into multiple sections to provide a narrative summary of the patient case-specific clinical and genetic findings and include suggestions of potential management or treatment strategies that may be relevant (see Supplementary Material S4 for a sample case report). It is critical to note that these AI-generated outputs, including the disease diagnosis and suggestions of management approaches, are provided for research and informational purposes only and are not a substitute for professional medical expert judgment or advice. To reinforce this, each report includes the following disclaimer: “Therapeutic Options: Therapeutic interventions must be determined by the patient’s treating physician. This report does not provide treatment prescriptions” (Supplementary Material S4). In addition, as a direct technical precaution, a prominent disclaimer is added to the top of every AI-reformatted case report on the platform, reading “Disclaimer: LLM-generated case reports may contain errors, hallucinations, or omissions. This report is strictly for informational and research purposes only and does not constitute medical advice or clinical guidance. Reader verification against the primary source is strongly advised”.

## 7. Discussion

Offering one of the largest virtual registries for PMDs, MSeqDR PMD-VR’s primary strength is its comprehensive breadth, achieved through semi-automated published literature mining and the integration of diverse data sources. This feature complements traditional registries built through slower, active patient enrollment, which excel in capturing deep, longitudinal clinical data and facilitating patient-centered research but may not be publicly accessible or readily searchable to facilitate ongoing research inquiries. In contrast, the MSeqDR PMD-VR prioritizes breadth and end-user-ready accessibility of published case-level data, offering a powerful and complementary resource for PMD research. It is a public resource and offers easy access to the PMD community, rather than being locked like most traditional registries, and additional publications and data from other PMD registries can be readily integrated in PMD-VR over time.

**Unprecedented Scale and Scope:** The PMD-VR has accumulated a large cohort of 11,000 individual cases across over 100 mitochondrial diseases that substantially surpasses most traditional single-disease phenotype registries. For LSS, PMD-VR hosts 2300 cases, exceeding the <200 cases in the CureMito Leigh Syndrome Registry [5] and broader PMD clinician-entered or patient-populated registries established worldwide to date. This large dataset provides unique statistical power for investigating genotype–phenotype correlations, symptom correlation, and disease epidemiology of these individually rare PMD subtypes. Overall, PMD-VR represents a significant addition to global mitochondrial disease community resources and is poised to make impactful contributions to the rare disease community focused on PMDs.

**A Versatile Bioinformatics and Curation Platform for Scalability and Broader Rare Disease Applications:** The registry’s construction is empowered by a custom-built, web-based and database-driven bioinformatics and curation platform for semi-automated literature case mining and efficient data processing, including potential capabilities to support future crowdsourced PMD case curation. This platform enables scalable data acquisition and standardization, which is crucial for maintaining and expanding the PMD-VR or adapting it for non-PMD virtual registries. Indeed, the platform’s modular design is versatile and can be adapted to build new custom virtual case registries from tabular data beyond mitochondrial disorders and integrate and disseminate their case-specific disease phenotype, gene, and variant associations. In one example, the UMDF_No_Cost_Genetic_Test dataset of one 335 case–cohort was captured with the PMD-VR platform without being published (Table 1). In addition, some components of the platform were customized and used to establish a case and sample portal for production-stage use at the Center for Personalized Medicine at Children’s Hospital Los Angeles, a genomic clinical diagnostic laboratory [23].

**AI-Empowered Knowledge Base and Clinical Insight Prototype on Affordable Hardware:** An ongoing innovation in development of PMD-VR is GenAI platform prototype integration, which can be run on personal laptops. This system integrates both a local mitochondrial disease knowledge base derived from the registry data and external biomedical knowledge sources to generate simulated “clinical visit reports” and citations to potential treatment suggestions based on individual case symptoms, genetics, the relevant public literature, clinical trial results, and drug information. Although this proof-of-concept prototype is currently focused only on user education purposes and not providing expert clinical advice, it illustrates the potential of generative AI to enhance phenotypic data interpretation and enable complex knowledge synthesis in virtual registries.

**Practical Utilization in ClinGen Expert Panels’ Variant/Gene Curation**: PMD-VR has already been used to effectively support the NIH-funded ClinGen Mitochondrial Disease Mito-GCEP [10] and Mito-VCEP [11] expert panels in their work to curate knowledge on published genes and variants associated with mitochondrial diseases. During some biocurator and expert review committee meetings, PMD-VR was used to quickly identify additional cases that resulted in increased population (PS1, PS4) scores to upgrade gene/variant classifications to “definitive” or “pathogenic” following ACMG guidelines. This usage highlights the registry’s direct contribution to fulfilling the objectives of the NIH-funded research program, whose scope includes knowledge dissemination to the mitochondrial community via development of PMD-VR.

**Addressing Limitations and Weaknesses:** This project has been run largely with volunteer effort, with limited resources available for comprehensive curation and data transformation. For example, only 2952 (25.5%) of the 11,000 cases have raw disease names mapped to 105 standardized OMIM terms so far. Therefore, cohort characterization analysis in this article is preliminary and must be interpreted with caution because characterization on most clinical features was based on curated small subsets of the whole registry’s cases, and the subsets may not always be identical for different features. So additional effort is required for registry data curation by developing a HiTL GenAI-based framework to speed up curation while reducing manual effort.

Inherent limitations exist for any virtual registry derived from literature mining. These include high data heterogeneity and variability in data quality across publications regarding reporting standards, missing clinical data, diagnostic criteria, inconsistent gene and phenotype/disease nomenclatures, limited genetic testing capabilities and/or ascertainment bias, and varied depth of phenotyping across studies and time periods. Despite substantial MSeqDR team efforts toward achieving consensus standardization, significant heterogeneity and untransformed data await further curation refinement.

Literature-based registries are susceptible to publication bias and case representativeness issues. Cases with novel disease symptoms and with positive molecular findings, or those studied in well-resourced centers, may be over-represented, while negative cases or subsequent cases of a published gene or variant or disorder might be less likely to be published. This could impact the severity or breath of representation of cases in the virtual registry and limit its generalizability to the broader PMD patient population. So, any epidemiological conclusions (such as age distributions or variant frequencies) derived from it are fragile and must be interpreted with caution against publication and reporting biases. There are potentially some duplicated cases if they were published in different studies under different contexts across time. Further data cleaning—potentially with AI aids—is required to identify and consolidate such duplicated cases. These factors must be considered when interpreting the cohort’s demographic, genetic, and clinical features.

GenAI tool limitation is another important factor. The LLMs speed up case data standardization and generate layman-friendly, narrative clinical reports. However, they showed variable hallucination rates that are most evident in HPO ID and PubMed ID assignments but also appear in other data mapping tasks. GenAI model selection, prompt optimization, and rigorous validation with conventional informatics methods can enable error rate reduction. An emerging risk for re-identification exists in the big data era, especially for very rare diseases with defining features. Furthermore, adherence to evolving regulatory requirements, such as GDPR in Europe and other data privacy measures, is critical and must be continuously addressed.

Future efforts will focus on expanding biocuration and refining the standardization of clinical phenotype data into established ontologies (HPO, MONDO, OMIM). To date, HPO mapping has been completed for approximately 4400 of the over 11,000 cases in the PMD-VR (including 3696 cases sourced from MitoPhen), a process limited by the significant human effort required for manual validation. Our GenAI-powered prototype has demonstrated highly reliable performance in standardizing age-related data and promising results in phenotypic mapping. Refining GenAI prompt engineering will further enhance case data mapping precision. Furthermore, as GenAI tools rapidly evolve, we will proactively evaluate and integrate optimal models for different tasks in virtual registry case data curation. For the knowledge base engine, future efforts will explore the integration of knowledge graphs (e.g., Neo4j), which excel at modeling the complex relationships between patients, diseases, and phenotypes. Rapidly evolving clinical and medical domain-specific LLMs will also be investigated. Single-case HPO mapping refinement can be conducted at our AI4HPO Clinical Phenotype Workbench (https://mseqdr.com/ai4hpo.php), though it is computationally intensive and therefore limited to small-scale workloads. Agentic AI will be explored for scaled case data processing, and specialized third-party AI tools like Python-based RAG-HPO [25], that leverages retrieval-augmented generation (RAG), may be explored for elevating the speed and accuracy of HPO term assignment within the PMD-VR.

Recognizing the complementary strengths of literature-derived virtual registries and traditional patient registries, active pursuit of collaborations and data-sharing partnerships is critical with existing clinician-entered PMD registries such as GENOMIT hosted in Europe (https://www.genomit.eu/) and patient-populated registries such as MitoShare hosted by the United Mitochondrial Disease Foundation (UMDF). The goal of these partnerships will be to develop and consolidate informatics standards, share resources, and harmonize nomenclature to facilitate large-scale data integration and accelerate research for the global mitochondrial disease community.

## 8. Conclusions

MSeqDR PMD-VR represents a significant advance built by the MSeqDR Consortium to meaningfully enhance community-accessible web resources that enable rare mitochondrial disease research and knowledge dissemination. Through leveraging the power of literature mining, expert manual curation, and cutting-edge generative AI technologies, PMD-VR offers an unprecedented breadth of case-level data and novel tools to the PMD research community. It has already meaningfully contributed to ClinGen Mito-GCEP gene–disease curation projects and a clinical diagnostic laboratory report generation process. Further curation that incorporates advanced AI tools to address platform limitations will unlock the potential of this virtual disease case-level registry to accelerate PMD research towards improved diagnostics, treatments, and outcomes for people of different origins, ages, phenotypes, and genotypes affected by PMD.

## Figures and Tables

**Table 1:** Ten datasets with the most cases per cohort used as sources for PMD virtual registry *.

PubMed ID/Cohort Name	Number of Cases	TITLE/Cohort Name
34428295	3696	34428295-MitoPhen database: a human phenotype ontology-based approach to identify mitochondrial DNA diseases
37255483	440	37255483-MITO_GCEP_Case_20230620 Expert panel curation of 113 primary mitochondrial disease genes for the Leigh syndrome spectrum
38703036	394	Primary mitochondrial disorders and mimics: Insights from a large French cohort
UMDF No Cost Genetic Test Cohort	335	UMDF_No_Cost_Genetic_Test Cohort
39533303	295	Genetic landscape of primary mitochondrial diseases in children and adults using molecular genetics and genomic investigations of mitochondrial and nuclear genome-Table mtDNA PMD
34490615	257	Prevalence and clinical prediction of mitochondrial disorders in a large neuropediatric cohort
39039281	252	Next-generation phenotyping integrated in a national framework for patients with ultrarare disorders improves genetic diagnostics and yields new molecular findings
34298071	226	Long-term prognosis and genetic background of cardiomyopathy in 223 pediatric mitochondrial disease patients
35094435	209	Leigh Syndrome: A Study of 209 Patients at the Beijing Children s Hospital; Table S3. HPO per case
38018320	155	The clinical, myopathological, and genetic analysis of 155 Chinese mitochondrial ophthalmoplegia patients with mitochondrial DNA single large deletions

*: The full dataset list is accessible through the PMD-VR case browser (Figure 2).

## Data Availability

The complete, harmonized case-level dataset generated and analyzed during this study is publicly available through the MSeqDR Consortium’s Primary Mitochondrial Disease Virtual Registry (PMD-VR), accessible at https://mseqdr.org/virtualregistry.php. This resource ensures broad accessibility for approved researchers, clinicians, and patient advocacy groups worldwide, fostering collaboration and data sharing within the global PMD community. Summaries of the dataset are provided in the main text and Supplementary Materials of this article. All source data were derived from public PubMed resources. Further inquiries regarding the data or methodology may be directed to the corresponding author upon reasonable request.

## Supplementary Materials

The following supporting information can be downloaded at: https://www.mdpi.com/article/10.3390/genes17070757/s1, Supplementary Figure S1. MSeqDR Mitochondrial Disease Virtual Registry (PMD-VR) case data upload interface. Supplementary Figure S2. Case raw phenotype-to-HPO mapping with conventional bioinformatics semantic similarity search tools. Supplementary Figure S3. PMD single-case tabular report example for MS01032740. Supplementary Table S1. Summary of some core data elements—raw term name mapping and cases. Supplementary Table S2. Raw data complexity in (A) disease, ethnicity, and genes and (B) mode of inheritance and mutation terms, reflecting the need for standardization. Supplementary Table S3. Abbreviation and full term correlation table. Supplementary Table S4. AI4Age sample report for age at onset, age at death, and survival time. Supplementary Table S5. Phenotype and HPO mapping for case MS01003000 Using Gemini-2.5-Pro API. Supplementary Materials S4 (in this file). GenAI-generated per-case report for case MS01003000.
